# Crystal structure of 5-[2-(9*H*-carbazol-9-yl)eth­yl]-1,3,4-oxa­diazole-2(3*H*)-thione

**DOI:** 10.1107/S2056989017009252

**Published:** 2017-06-23

**Authors:** Jim Simpson, Shaaban K. Mohamed, Talaat I. El-Emary, Mustafa R. Albayati

**Affiliations:** a Department of Chemistry, University of Otago, PO Box 56, Dunedin, New Zealand; b Chemistry and Environmental Division, Manchester Metropolitan University, Manchester M1 5GD, England; c Chemistry Department, Faculty of Science, Minia University, 61519 El-Minia, Egypt; d Department of Chemistry, Faculty of Science, Assiut University, 71515 Assiut, Egypt; e Kirkuk University, College of Education, Department of Chemistry, Kirkuk, Iraq

**Keywords:** crystal structure, carbazole, oxa­diazo­lethione, hydrogen bonds, C—H⋯π(ring) contacts, π–π stacking

## Abstract

The title compound, C_16_H_13_N_3_OS, comprises an oxa­diazo­lethione ring bound to the N atom of an almost planar carbazole ring system (r.m.s. deviation = 0.0088 Å) through an ethyl­ene chain. The oxa­diazole ring is inclined to the the carbazole ring system by 40.71 (6)°. In the crystal, N—H⋯O, N—H⋯S, C—H⋯N and C—H⋯S hydrogen bonds combine with C—H⋯π(ring) and π–π contacts to stack the mol­ecules along the *b*-axis direction.

## Abstract 2

The mol­ecular and crystal structure of an unusual oxa­diazo­lethione derivative is reported. The crystal structure is stabilized by N–H⋯O, N–H⋯S, C–H⋯N and C–H⋯S hydrogen bonds together with C–H⋯π(ring) and π–π contacts.

## Chemical context   

Carbazole derivatives have been shown to have several industrial applications including use in optoelectronic devices (Fitilis *et al.*, 2007 ▸; Peng *et al.*, 2011 ▸), dye-sensitized solar cells (Li *et al.*, 2010 ▸) and photochromic dyes (Billah *et al.*, 2008 ▸). Moreover, fused heterocycles with carbazole scaffolds are noted for their biological activities. They are found in drugs such as tubingensin A and B and have been shown to have both anti­viral and cytotoxic activities (TePaske *et al.*, 1989 ▸). The anti-inflammatory agents caprofen and etodolaca and the anti­pyretic agent nincazole (Ghoneim *et al.*, 2006 ▸) are also carbazole based. The biological activity of so many carbazole-based heterocycles encouraged us to synthesize the title compound and its mol­ecular crystal structure is reported here.
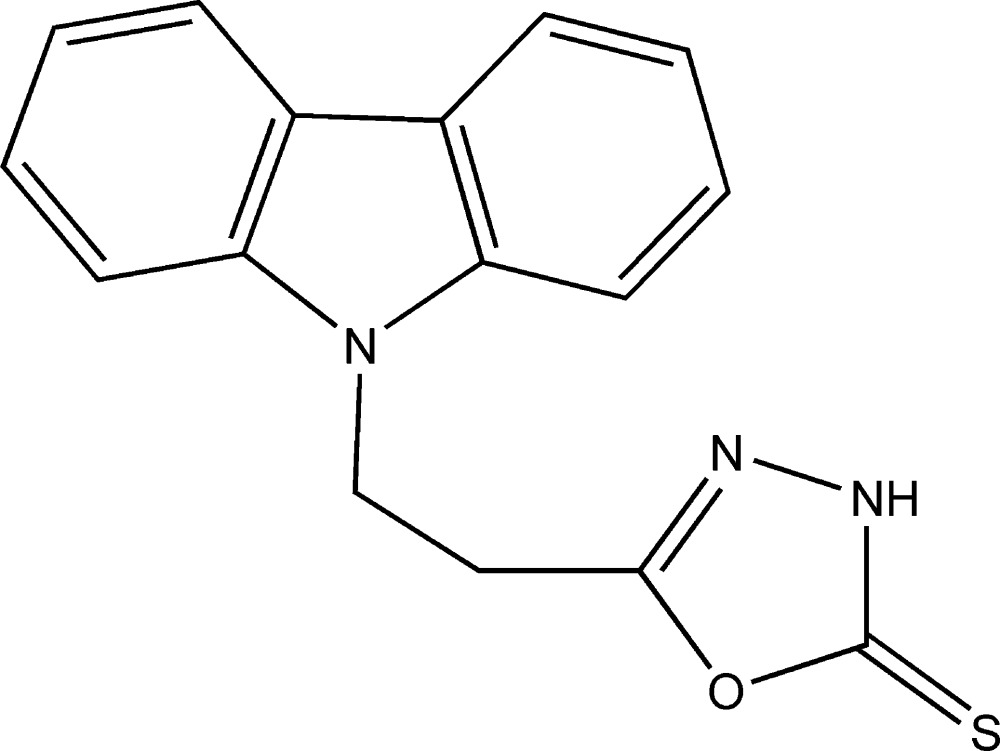


## Structural commentary   

**Figure 1:**
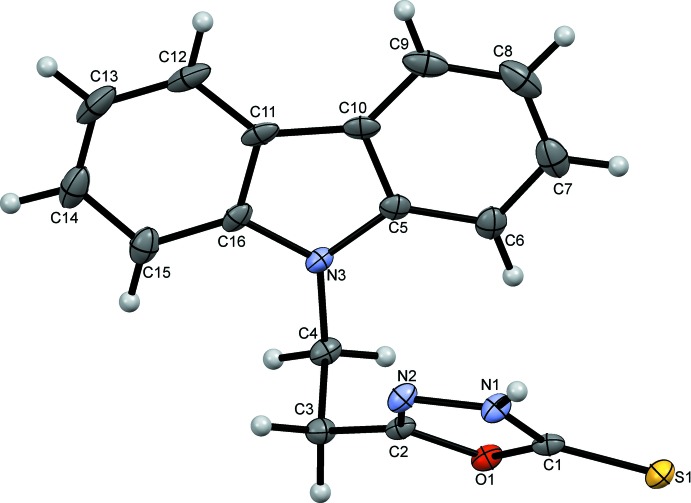
The mol­ecular structure of (I) with ellipsoids drawn at the 50% probability level.

In the title compound C_16_H_13_N_3_OS, (I), the oxa­diazo­lethione ring binds to the carbazole ring system through a C2–C3–C4–N3 ethyl­ene chain with the ring systems inclined at an angle of 40.71 (6)°, Fig. 1 ▸. The carbazole ring system is almost planar with the outer C5–C10 and C11–C16 benzene rings subtending angles of 0.38 (13) and 0.64 (13)°, respectively, to the central N3/C5/C10/C11/C16 ring. Bond lengths and angles in both ring systems are normal and similar to those found in the numerous other carbazole structures (see, for example, Kimura *et al.*, 1985 ▸) and those of the few known oxa­diazo­lethione derivatives with alkane chains at C5 (Khan *et al.* 2014 ▸; Zheng *et al.* 2006 ▸).

## Supra­molecular features   

**Figure 2:**
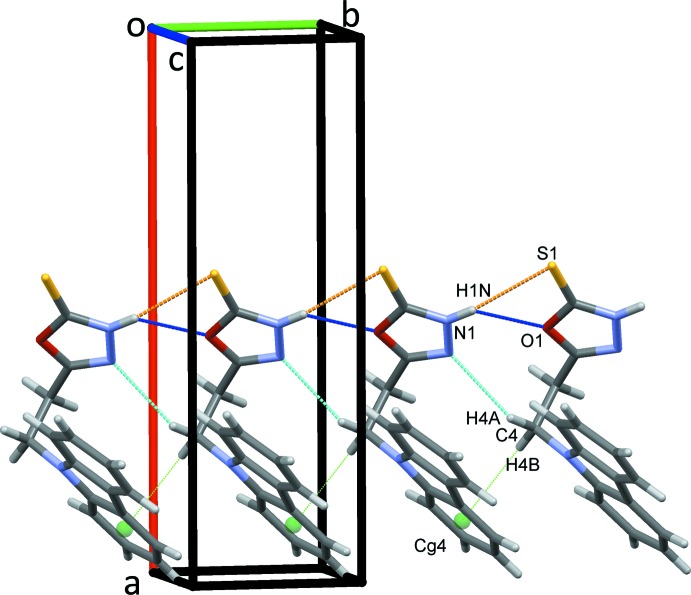
Rows of mol­ecules of (I) along *b*. In this and subsequent figures, N—H⋯S (orange), N—H⋯O (dark blue) and C—H⋯N (light blue) hydrogen bonds are drawn as coloured dashed lines. C—H⋯π contacts are shown as green dotted lines with ring centroids displayed as coloured spheres.

**Figure 3:**
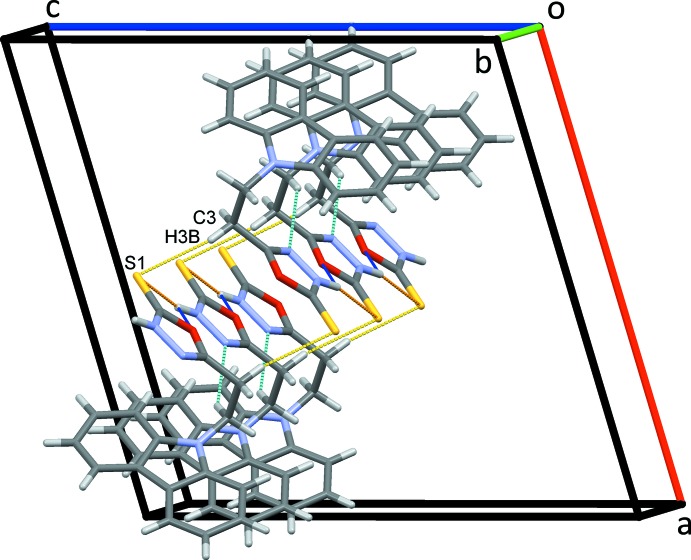
Inversion dimers formed by C—H⋯S hydrogen bonds (dashed yellow lines) stacking rows of mol­ecules of (I) along *b*.

**Figure 4:**
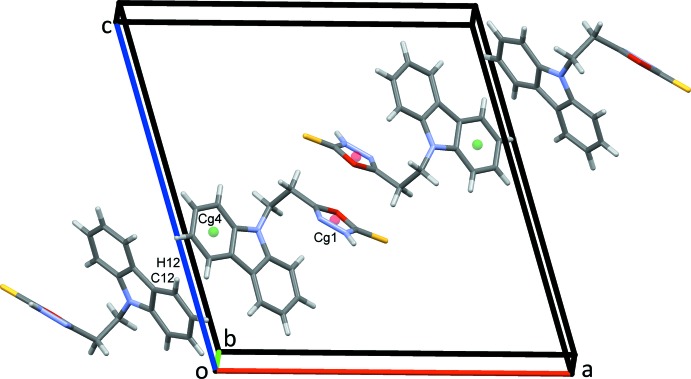
Chains of mol­ecules of (I) along the *ac* diagonal. Centroid–centroid contacts are drawn as green dotted lines.

**Figure 5:**
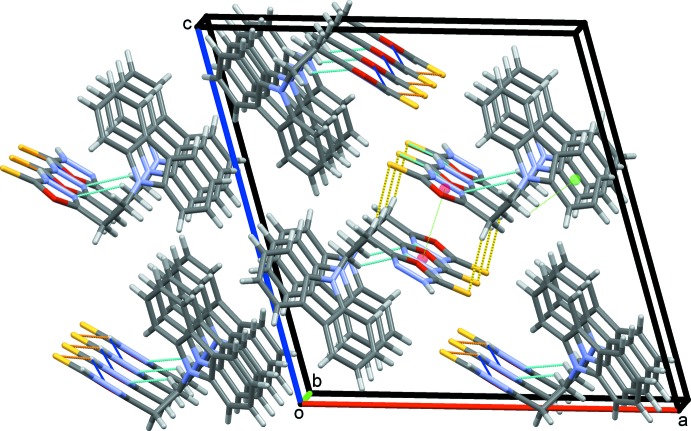
Overall packing of (I) viewed along the *b*-axis direction. Representative C—H⋯π hydrogen bonds and π–π contacts are shown as green dotted lines.

In the crystal, classical N1—H1*N*⋯O1 and N1—H1*N*⋯S1 hydrogen bonds form *C*(4) chains of mol­ecules linked in a head-to-head fashion along the *b-*axis direction, Fig. 2 ▸. These contacts are bolstered by the C4 atom acting as a bifurcated donor forming weaker C4—H4*A*⋯N2 hydrogen bonds and C4—H4*B*⋯*Cg*4 inter­actions, Table 1 ▸. In the chains, the mean plane of the oxa­diazole ring is inclined at 10.7° to (101). The N—H⋯O and N—H⋯S hydrogen bonds also impose close O1⋯N2(*x*, *y* − 1, *z*) contacts of 2.9516 (18) Å. Adjacent chains are further linked by C3—H3*B*⋯S1 hydrogen bonds that form inversion dimers, enclosing 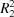
(12) rings. This combination of contacts stacks mol­ecules along the *b-*axis direction, Fig. 3 ▸. Adjacent oxa­diazole rings form dimers through *Cg*1⋯*Cg*1^vi^ π–π contacts with centroid-to-centroid separations of 3.3931 (9) Å *Cg*1 is the centroid of the O1/C2/N3/N4/C5 ring; symmetry code: (vi) 1 − *x*, 1 − *y*, 1 − *z*]. These dimers are linked by much weaker C12—H12⋯*Cg*4 inter­actions, Table 1 ▸, forming chains along the *ac* diagonal, Fig. 4 ▸. This substantial array of contacts combines to form a three-dimensional network structure, Fig. 5 ▸.

## Database survey   

Structures of carbazole derivatives abound in the Cambridge Structural Database (Version 5.38, November 2016 with one update; Groom *et al.*, 2016 ▸) with 428 hits for solely organic mol­ecules. Those with alkane chain substituents, at least two carbon atoms long on the pyrrole N atom, are less abundant with 47 hits for organic mol­ecules alone. The simplest of these is *N*-ethyl carbazole itself (Kimura *et al.*, 1985 ▸). This compound in fact appears in a number of manifestations as it seems to readily form co-crystals (Lee & Wallwork, 1978 ▸; Hosomi *et al.*, 2000 ▸; Matsuoka *et al.*, 1988 ▸; Zhu *et al.*, 2014 ▸). No examples were found of oxa­diazole rings at the end of the alkane chains; indeed, the only derivatives with simple five-membered rings in that position were dioxaborolane derivatives (Kalinin *et al.*, 2003 ▸; Geier *et al.*, 2009 ▸). In contrast, 1,3,4-oxa­diazole-2-thio­nes are far less abundant with only 29 unique organic structures reported. Furthermore, crystal structures of compounds with a chain of two or more methyl­ene units bound to the 5-carbon are rare, with only three such structures found: 5-[2-(2-meth­oxy­phen­yl)eth­yl]-1,3,4-oxa­diazole-2(3*H*)-thione and 5-[2-(4-meth­oxy­phen­yl)eth­yl]-1,3,4-oxa­diazole-2(3*H*)-thione (Khan *et al.* 2014 ▸) and 5-[3-(quinolin-8-yl­oxy)prop­yl]-1,3,4-oxa­diazole-2(3*H*)-thione (Zheng *et al.* 2006 ▸)

## Synthesis and crystallization   

A mixture of 3-(9*H*-carbazol-9-yl)propane­hydrazide (1.09 g, 4 mmol) and carbon di­sulfide (3 ml) in pyridine (15 mL) was heated under reflux on a water bath (333–343 K) overnight. The excess carbon di­sulfide was removed under reduced pressure and the reaction mixture was then poured into ice-cold water. The resulting precipitate was collected by filtration, washed with water, dried and recrystallized from mixed solvents of dioxane–water (1:1) to give (I) in 66% yield; m.p. 469–471 K. IR: NH, 3197, CH aromatic 3050, CH aliphatic 2940 cm^−1^. ^1^H NMR: δ (ppm) (DMSO-*d*_6_) 2.35 (*t*, 2H, CH2), 4.12 (*t*, 2H, CH2), 7.35–8.38 (*m*, 8H, Ar-H), 9.95 (*s*, 1H, NH). ^13^C NMR (100 MHz, DMSO-*d*_6_, DEPT) δ (ppm): 34.9, 51.4, 109.6, 119.9, 121.4, 122.8, 156.8, 188.9. ms: *m*/*z* 295 (*M* ^+^) as mol­ecular ion peak and base peak. Analysis calculated for C_16_ H_13_ N_3_OS (295.4): C, 65.06; H, 4.44; N, 5.42. Found: C, 65.38; H, 4.65; N, 5.48.

## Refinement   

Crystal data, data collection and structure refinement details are summarized in Table 2 ▸. The N-bound hydrogen atom was located in a difference-Fourier map and its coordinates refined with *U*_iso_ = 1.2*U*_eq_ (N). All H atoms bound to C were refined using a riding model with *d*(C—H) = 0.95 Å and *U*_iso_(H) = 1.2*U*_eq_(C) for aromatic, *d*(C—H) = 0.99 Å and *U*_iso_(H) = 1.2*U*_eq_(C) for CH_2_ H atoms.

## Supplementary Material

Crystal structure: contains datablock(s) global, I. DOI: 10.1107/S2056989017009252/mw2132sup1.cif

Structure factors: contains datablock(s) I. DOI: 10.1107/S2056989017009252/mw2132Isup2.hkl

Supporting information file. DOI: 10.1107/S2056989017009252/mw2132Isup3.cml

CCDC reference: 1557216

Additional supporting information:  crystallographic information; 3D view; checkCIF report

## Figures and Tables

**Table 1:** Hydrogen-bond geometry (Å, °) *Cg*4 is the centroid of the C11–C16 ring.

*D*—H⋯*A*	*D*—H	H⋯*A*	*D*⋯*A*	*D*—H⋯*A*
N1—H1*N*⋯S1^i^	0.89 (2)	2.75 (2)	3.6053 (14)	162.8 (19)
N1—H1*N*⋯O1^i^	0.89 (2)	2.62 (2)	3.0707 (18)	112.5 (16)
C3—H3*B*⋯S1^ii^	0.99	2.93	3.9061 (16)	169
C4—H4*A*⋯N2^iii^	0.99	2.67	3.495 (2)	141
C4—H4*B*⋯*Cg*4^iii^	0.99	2.87	3.4577 (17)	119
C12—H12⋯*Cg*4^i^	0.95	3.22	4.073 (2)	151

Symmetry codes: (i) 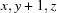; (ii) 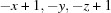; (iii) 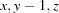.

**Table 2:** Experimental details

Crystal data	
Chemical formula	C_16_H_13_N_3_OS
*M*_r_	295.35
Crystal system, space group	Monoclinic, *P*2_1_/*c*
Temperature (K)	100
*a*, *b*, *c* (Å)	16.6868 (5), 4.9600 (1), 17.2353 (6)
β (°)	105.909 (3)
*V* (Å^3^)	1371.87 (7)
*Z*	4
Radiation type	Cu *K*α
μ (mm^−1^)	2.11
Crystal size (mm)	0.27 × 0.15 × 0.09

Data collection	
Diffractometer	Agilent SuperNova, Dual, Cu at zero, Atlas
Absorption correction	Multi-scan (*CrysAlis PRO*; Agilent, 2014 ▸)
*T*_min_, *T*_max_	0.763, 1.000
No. of measured, independent and observed [*I* > 2σ(*I*)] reflections	11013, 2849, 2626
*R*_int_	0.063
(sin θ/λ)_max_ (Å^−1^)	0.631

Refinement	
*R*[*F* ^2^ > 2σ(*F* ^2^)], *wR*(*F* ^2^), *S*	0.045, 0.125, 1.06
No. of reflections	2849
No. of parameters	193
H-atom treatment	H atoms treated by a mixture of independent and constrained refinement
Δρ_max_, Δρ_min_ (e Å^−3^)	0.43, −0.48

Computer programs: *CrysAlis PRO* (Agilent, 2014 ▸), *SHELXT* (Sheldrick, 2015*a* ▸), *SHELXL2014* (Sheldrick, 2015*b* ▸), *TITAN2000* (Hunter & Simpson, 1999 ▸), *Mercury* (Macrae *et al.*, 2008 ▸), *enCIFer* (Allen *et al.*, 2004 ▸), *PLATON* (Spek, 2009 ▸), *publCIF* (Westrip, 2010 ▸) and *WinGX* (Farrugia, 2012 ▸).

## supplementary crystallographic information

### Crystal data

**Table:**

C_16_H_13_N_3_OS	*F*(000) = 616
*M**_r_* = 295.35	*D*_x_ = 1.430 Mg m^−^^3^
Monoclinic, *P*2_1_/*c*	Cu *K*α radiation, λ = 1.54184 Å
*a* = 16.6868 (5) Å	Cell parameters from 7352 reflections
*b* = 4.9600 (1) Å	θ = 6.5–76.5°
*c* = 17.2353 (6) Å	µ = 2.11 mm^−^^1^
β = 105.909 (3)°	*T* = 100 K
*V* = 1371.87 (7) Å^3^	Plate, colourless
*Z* = 4	0.27 × 0.15 × 0.09 mm

### Data collection

**Table:**

Agilent SuperNova, Dual, Cu at zero, Atlas diffractometer	2849 independent reflections
Radiation source: SuperNova (Cu) X-ray Source	2626 reflections with *I* > 2σ(*I*)
Detector resolution: 5.1725 pixels mm^-1^	*R*_int_ = 0.063
ω scans	θ_max_ = 76.5°, θ_min_ = 5.3°
Absorption correction: multi-scan (*CrysAlis PRO*; Agilent, 2014)	*h* = −20→20
*T*_min_ = 0.763, *T*_max_ = 1.000	*k* = −4→6
11013 measured reflections	*l* = −20→21

### Refinement

**Table:**

Refinement on *F*^2^	0 restraints
Least-squares matrix: full	Hydrogen site location: mixed
*R*[*F*^2^ > 2σ(*F*^2^)] = 0.045	H atoms treated by a mixture of independent and constrained refinement
*wR*(*F*^2^) = 0.125	*w* = 1/[σ^2^(*F*_o_^2^) + (0.079*P*)^2^ + 0.5548*P*] where *P* = (*F*_o_^2^ + 2*F*_c_^2^)/3
*S* = 1.06	(Δ/σ)_max_ = 0.001
2849 reflections	Δρ_max_ = 0.43 e Å^−^^3^
193 parameters	Δρ_min_ = −0.48 e Å^−^^3^

### Special details

**Table:**

Geometry. All esds (except the esd in the dihedral angle between two l.s. planes) are estimated using the full covariance matrix. The cell esds are taken into account individually in the estimation of esds in distances, angles and torsion angles; correlations between esds in cell parameters are only used when they are defined by crystal symmetry. An approximate (isotropic) treatment of cell esds is used for estimating esds involving l.s. planes.

### Fractional atomic coordinates and isotropic or equivalent isotropic displacement parameters (Å^2^)

**Table:**

	*x*	*y*	*z*	*U*_iso_*/*U*_eq_	
O1	0.55153 (7)	0.1963 (2)	0.58574 (7)	0.0155 (3)	
C1	0.50674 (9)	0.3454 (3)	0.62638 (9)	0.0151 (3)	
S1	0.43636 (2)	0.21061 (9)	0.66526 (2)	0.01955 (16)	
N1	0.53275 (8)	0.5979 (3)	0.62294 (8)	0.0158 (3)	
H1N	0.5152 (14)	0.740 (5)	0.6449 (14)	0.019*	
N2	0.59279 (8)	0.6190 (3)	0.58088 (8)	0.0168 (3)	
C2	0.60197 (9)	0.3745 (3)	0.56023 (9)	0.0146 (3)	
C3	0.66006 (10)	0.2674 (3)	0.51646 (10)	0.0175 (3)	
H6A	0.6797	0.4173	0.4885	0.021*	
H3B	0.6301	0.1372	0.4751	0.021*	
C4	0.73573 (9)	0.1269 (3)	0.57370 (10)	0.0168 (3)	
H4A	0.7160	−0.0214	0.6022	0.020*	
H4B	0.7707	0.0470	0.5416	0.020*	
N3	0.78545 (8)	0.3109 (3)	0.63217 (8)	0.0151 (3)	
C5	0.77515 (9)	0.3625 (3)	0.70793 (9)	0.0154 (3)	
C6	0.72386 (10)	0.2314 (4)	0.74787 (10)	0.0201 (4)	
H6	0.6892	0.0851	0.7237	0.024*	
C7	0.72578 (11)	0.3239 (4)	0.82431 (11)	0.0273 (4)	
H7	0.6917	0.2390	0.8531	0.033*	
C8	0.77677 (13)	0.5393 (4)	0.85996 (10)	0.0309 (4)	
H8	0.7763	0.5987	0.9122	0.037*	
C9	0.82767 (11)	0.6669 (4)	0.82050 (11)	0.0262 (4)	
H9	0.8621	0.8130	0.8452	0.031*	
C10	0.82777 (9)	0.5777 (3)	0.74355 (10)	0.0182 (3)	
C11	0.87157 (9)	0.6589 (3)	0.68573 (10)	0.0190 (3)	
C12	0.93139 (10)	0.8561 (4)	0.68605 (12)	0.0274 (4)	
H12	0.9509	0.9717	0.7312	0.033*	
C13	0.96158 (11)	0.8797 (4)	0.61920 (13)	0.0327 (5)	
H13	1.0023	1.0130	0.6188	0.039*	
C14	0.93329 (11)	0.7113 (4)	0.55235 (13)	0.0296 (4)	
H14	0.9556	0.7320	0.5076	0.036*	
C15	0.87311 (10)	0.5133 (4)	0.54963 (11)	0.0223 (4)	
H15	0.8535	0.3998	0.5040	0.027*	
C16	0.84324 (9)	0.4908 (3)	0.61765 (10)	0.0167 (3)	

### Atomic displacement parameters (Å^2^)

**Table:**

	*U*^11^	*U*^22^	*U*^33^	*U*^12^	*U*^13^	*U*^23^
O1	0.0145 (5)	0.0103 (5)	0.0207 (6)	−0.0003 (4)	0.0030 (4)	−0.0018 (4)
C1	0.0127 (7)	0.0132 (7)	0.0162 (7)	0.0011 (6)	−0.0014 (5)	−0.0011 (5)
S1	0.0185 (2)	0.0185 (3)	0.0215 (2)	−0.00502 (14)	0.00544 (16)	−0.00070 (14)
N1	0.0149 (6)	0.0108 (7)	0.0222 (6)	−0.0002 (5)	0.0057 (5)	−0.0018 (5)
N2	0.0139 (6)	0.0136 (7)	0.0230 (7)	−0.0003 (5)	0.0052 (5)	0.0000 (5)
C2	0.0116 (6)	0.0129 (7)	0.0166 (7)	−0.0004 (6)	−0.0006 (5)	0.0005 (6)
C3	0.0155 (7)	0.0185 (8)	0.0173 (7)	0.0004 (6)	0.0023 (6)	−0.0030 (6)
C4	0.0136 (7)	0.0144 (7)	0.0217 (7)	0.0006 (6)	0.0035 (6)	−0.0029 (6)
N3	0.0110 (6)	0.0156 (7)	0.0184 (7)	−0.0021 (5)	0.0035 (5)	−0.0013 (5)
C5	0.0114 (6)	0.0162 (8)	0.0167 (7)	0.0050 (6)	0.0006 (5)	0.0015 (6)
C6	0.0144 (7)	0.0226 (8)	0.0231 (8)	0.0053 (6)	0.0047 (6)	0.0050 (6)
C7	0.0261 (9)	0.0351 (11)	0.0225 (9)	0.0143 (8)	0.0097 (7)	0.0099 (7)
C8	0.0371 (10)	0.0367 (11)	0.0166 (8)	0.0175 (8)	0.0035 (7)	0.0002 (7)
C9	0.0254 (8)	0.0242 (9)	0.0215 (8)	0.0089 (7)	−0.0061 (6)	−0.0032 (7)
C10	0.0131 (7)	0.0175 (8)	0.0188 (7)	0.0048 (6)	−0.0047 (5)	0.0011 (6)
C11	0.0098 (6)	0.0157 (8)	0.0259 (8)	0.0017 (6)	−0.0048 (6)	0.0030 (6)
C12	0.0145 (7)	0.0189 (9)	0.0395 (10)	−0.0035 (7)	−0.0081 (7)	0.0056 (8)
C13	0.0122 (7)	0.0283 (10)	0.0525 (12)	−0.0042 (7)	−0.0001 (7)	0.0169 (9)
C14	0.0167 (8)	0.0314 (11)	0.0424 (11)	0.0031 (7)	0.0107 (7)	0.0164 (8)
C15	0.0173 (7)	0.0217 (9)	0.0295 (8)	0.0040 (6)	0.0090 (6)	0.0063 (7)
C16	0.0089 (6)	0.0152 (8)	0.0247 (8)	0.0025 (5)	0.0024 (5)	0.0048 (6)

### Geometric parameters (Å, º)

**Table:**

O1—C1	1.3723 (18)	C6—C7	1.387 (3)
O1—C2	1.3732 (18)	C6—H6	0.9500
C1—N1	1.332 (2)	C7—C8	1.399 (3)
C1—S1	1.6452 (16)	C7—H7	0.9500
N1—N2	1.3922 (18)	C8—C9	1.379 (3)
N1—H1N	0.89 (2)	C8—H8	0.9500
N2—C2	1.285 (2)	C9—C10	1.398 (2)
N2—O1^i^	2.9516 (18)	C9—H9	0.9500
C2—C3	1.480 (2)	C10—C11	1.445 (2)
C3—C4	1.540 (2)	C11—C12	1.396 (2)
C3—H6A	0.9900	C11—C16	1.411 (2)
C3—H3B	0.9900	C12—C13	1.383 (3)
C4—N3	1.442 (2)	C12—H12	0.9500
C4—H4A	0.9900	C13—C14	1.396 (3)
C4—H4B	0.9900	C13—H13	0.9500
N3—C16	1.386 (2)	C14—C15	1.396 (3)
N3—C5	1.387 (2)	C14—H14	0.9500
C5—C6	1.397 (2)	C15—C16	1.398 (2)
C5—C10	1.411 (2)	C15—H15	0.9500
			
C1—O1—C2	106.49 (12)	C7—C6—H6	121.4
N1—C1—O1	104.73 (13)	C5—C6—H6	121.4
N1—C1—S1	132.68 (13)	C6—C7—C8	121.53 (17)
O1—C1—S1	122.56 (12)	C6—C7—H7	119.2
C1—N1—N2	112.56 (13)	C8—C7—H7	119.2
C1—N1—H1N	125.4 (14)	C9—C8—C7	121.11 (17)
N2—N1—H1N	122.1 (14)	C9—C8—H8	119.4
C2—N2—N1	103.34 (13)	C7—C8—H8	119.4
C2—N2—O1^i^	165.93 (11)	C8—C9—C10	118.93 (18)
N1—N2—O1^i^	81.45 (9)	C8—C9—H9	120.5
N2—C2—O1	112.87 (13)	C10—C9—H9	120.5
N2—C2—C3	128.62 (15)	C9—C10—C5	119.30 (16)
O1—C2—C3	118.48 (14)	C9—C10—C11	134.28 (17)
C2—C3—C4	111.86 (13)	C5—C10—C11	106.41 (14)
C2—C3—H6A	109.2	C12—C11—C16	119.57 (17)
C4—C3—H6A	109.2	C12—C11—C10	133.50 (17)
C2—C3—H3B	109.2	C16—C11—C10	106.93 (14)
C4—C3—H3B	109.2	C13—C12—C11	118.63 (19)
H6A—C3—H3B	107.9	C13—C12—H12	120.7
N3—C4—C3	112.02 (13)	C11—C12—H12	120.7
N3—C4—H4A	109.2	C12—C13—C14	121.24 (17)
C3—C4—H4A	109.2	C12—C13—H13	119.4
N3—C4—H4B	109.2	C14—C13—H13	119.4
C3—C4—H4B	109.2	C15—C14—C13	121.73 (18)
H4A—C4—H4B	107.9	C15—C14—H14	119.1
C16—N3—C5	108.85 (13)	C13—C14—H14	119.1
C16—N3—C4	125.23 (14)	C14—C15—C16	116.51 (17)
C5—N3—C4	125.38 (13)	C14—C15—H15	121.7
N3—C5—C6	128.95 (15)	C16—C15—H15	121.7
N3—C5—C10	109.05 (14)	N3—C16—C15	128.93 (16)
C6—C5—C10	122.00 (15)	N3—C16—C11	108.74 (14)
C7—C6—C5	117.12 (17)	C15—C16—C11	122.32 (16)
			
C2—O1—C1—N1	−0.16 (15)	C8—C9—C10—C5	0.8 (2)
C2—O1—C1—S1	178.36 (11)	C8—C9—C10—C11	−179.84 (17)
O1—C1—N1—N2	0.37 (16)	N3—C5—C10—C9	179.65 (14)
S1—C1—N1—N2	−177.93 (12)	C6—C5—C10—C9	−1.3 (2)
C1—N1—N2—C2	−0.44 (17)	N3—C5—C10—C11	0.15 (17)
C1—N1—N2—O1^i^	166.09 (12)	C6—C5—C10—C11	179.21 (14)
N1—N2—C2—O1	0.32 (17)	C9—C10—C11—C12	1.0 (3)
O1^i^—N2—C2—O1	−108.2 (4)	C5—C10—C11—C12	−179.56 (18)
N1—N2—C2—C3	−177.54 (14)	C9—C10—C11—C16	−179.05 (17)
O1^i^—N2—C2—C3	73.9 (5)	C5—C10—C11—C16	0.35 (17)
C1—O1—C2—N2	−0.11 (17)	C16—C11—C12—C13	−0.3 (2)
C1—O1—C2—C3	177.99 (13)	C10—C11—C12—C13	179.55 (17)
N2—C2—C3—C4	102.47 (19)	C11—C12—C13—C14	0.1 (3)
O1—C2—C3—C4	−75.29 (17)	C12—C13—C14—C15	0.4 (3)
C2—C3—C4—N3	−62.96 (18)	C13—C14—C15—C16	−0.6 (3)
C3—C4—N3—C16	−79.78 (18)	C5—N3—C16—C15	179.80 (15)
C3—C4—N3—C5	90.85 (18)	C4—N3—C16—C15	−8.3 (3)
C16—N3—C5—C6	−179.58 (15)	C5—N3—C16—C11	0.82 (17)
C4—N3—C5—C6	8.5 (3)	C4—N3—C16—C11	172.75 (14)
C16—N3—C5—C10	−0.60 (17)	C14—C15—C16—N3	−178.53 (16)
C4—N3—C5—C10	−172.52 (14)	C14—C15—C16—C11	0.3 (2)
N3—C5—C6—C7	179.70 (15)	C12—C11—C16—N3	179.20 (14)
C10—C5—C6—C7	0.8 (2)	C10—C11—C16—N3	−0.72 (17)
C5—C6—C7—C8	0.0 (2)	C12—C11—C16—C15	0.1 (2)
C6—C7—C8—C9	−0.5 (3)	C10—C11—C16—C15	−179.78 (14)
C7—C8—C9—C10	0.0 (3)		

Symmetry code: (i) *x*, *y*+1, *z*.

### Hydrogen-bond geometry (Å, º)

Cg4 is the centroid of the C11–C16 ring.

**Table:**

*D*—H···*A*	*D*—H	H···*A*	*D*···*A*	*D*—H···*A*
N1—H1*N*···S1^i^	0.89 (2)	2.75 (2)	3.6053 (14)	162.8 (19)
N1—H1*N*···O1^i^	0.89 (2)	2.62 (2)	3.0707 (18)	112.5 (16)
C3—H3*B*···S1^ii^	0.99	2.93	3.9061 (16)	169
C4—H4*A*···N2^iii^	0.99	2.67	3.495 (2)	141
C4—H4*B*···*Cg*4^iii^	0.99	2.87	3.4577 (17)	119
C12—H12···*Cg*4^i^	0.95	3.22	4.073 (2)	151

Symmetry codes: (i) *x*, *y*+1, *z*; (ii) −*x*+1, −*y*, −*z*+1; (iii) *x*, *y*−1, *z*.
